# Adenoid cystic carcinoma of the breast: a case report and literature review

**DOI:** 10.1093/omcr/omag089

**Published:** 2026-06-08

**Authors:** Yassine Hamdaoui, Anass Elachchi, Cherifa Fekkoul, Hassane Ait Ali, Ayoub Kharkhach, Tariq Bouhout, Badr Serji

**Affiliations:** Faculty of Medicine and Pharmacy, Mohammed First University, BP 724 Hay Al Quods, Oujda 60000, Morocco; Department of Surgical Oncology, Oncology Hospital, Mohammed VI University Hospital, BP 4806 Oujda Universite 60049, Morocco; Faculty of Medicine and Pharmacy, Mohammed First University, BP 724 Hay Al Quods, Oujda 60000, Morocco; Department of Surgical Oncology, Oncology Hospital, Mohammed VI University Hospital, BP 4806 Oujda Universite 60049, Morocco; Faculty of Medicine and Pharmacy, Mohammed First University, BP 724 Hay Al Quods, Oujda 60000, Morocco; Department Radiotherapy, Oncology Hospital, Mohammed VI University Hospital, BP 4806 Oujda Universite 60049, Morocco; Faculty of Medicine and Pharmacy, Mohammed First University, BP 724 Hay Al Quods, Oujda 60000, Morocco; Department of Surgical Oncology, Oncology Hospital, Mohammed VI University Hospital, BP 4806 Oujda Universite 60049, Morocco; Faculty of Medicine and Pharmacy, Mohammed First University, BP 724 Hay Al Quods, Oujda 60000, Morocco; Department of Surgical Oncology, Oncology Hospital, Mohammed VI University Hospital, BP 4806 Oujda Universite 60049, Morocco; Faculty of Medicine and Pharmacy, Mohammed First University, BP 724 Hay Al Quods, Oujda 60000, Morocco; Department of Surgical Oncology, Oncology Hospital, Mohammed VI University Hospital, BP 4806 Oujda Universite 60049, Morocco; Faculty of Medicine and Pharmacy, Mohammed First University, BP 724 Hay Al Quods, Oujda 60000, Morocco; Department of Surgical Oncology, Oncology Hospital, Mohammed VI University Hospital, BP 4806 Oujda Universite 60049, Morocco

**Keywords:** adenoid cystic carcinoma, breast cancer, lumpectomy, radiotherapy, rare tumor

## Abstract

Adenoid cystic carcinoma (ACC) of the breast is an exceptionally rare malignancy, accounting for less than 0.1% of all breast cancer cases. It typically affects women between the ages of 50 and 60 and most commonly presents as a subareolar mass or breast pain. Radiological findings are often non-specific; however, diagnosis can be established through fine-needle aspiration cytology. In this report, we present the case of a 69-year-old woman who presented with pain in the outer quadrants of the right breast. Lumpectomy revealed adenoid cystic carcinoma, and the patient subsequently received postoperative radiotherapy. At 20 months of follow-up, no signs of recurrence or metastasis were observed. In conclusion, mammary ACC is a rare form of breast cancer. While surgical excision remains the cornerstone of treatment, the optimal approach to adjuvant therapy remains unclear due to the tumor’s rarity.

## Introduction

Adenoid cystic carcinoma (ACC), formerly known as cylindroma, is a malignant tumor that primarily affects the salivary glands and upper airways. Its occurrence in the breast is rare, accounting for less than 1% of all breast cancers [1, 2]. Compared to other sites, breast ACC has a particularly favorable prognosis [3, 4]. This paper presents a case of breast ACC and analyzes its epidemiological, clinical, therapeutic, and prognostic characteristics.

## Case report

A 69-year-old postmenopausal woman, mother of five children, with a history of hypertension, presented with a painful nodule in the outer quadrants of her right breast that had been progressively increasing in size over the past four months.

The patient was in good general health. Clinical examination revealed a 30 mm × 30 mm mass in the right breast at the junction of the outer quadrants, mobile relative to both the skin and the underlying muscle, with no inflammatory changes. No abnormalities were noted in the left breast, nor in the axillary or supraclavicular lymph nodes bilaterally.

Based on these findings, the tumor was staged as cT2N0M0, according to the AJCC/UICC 8th Edition.

**Figure 1:**
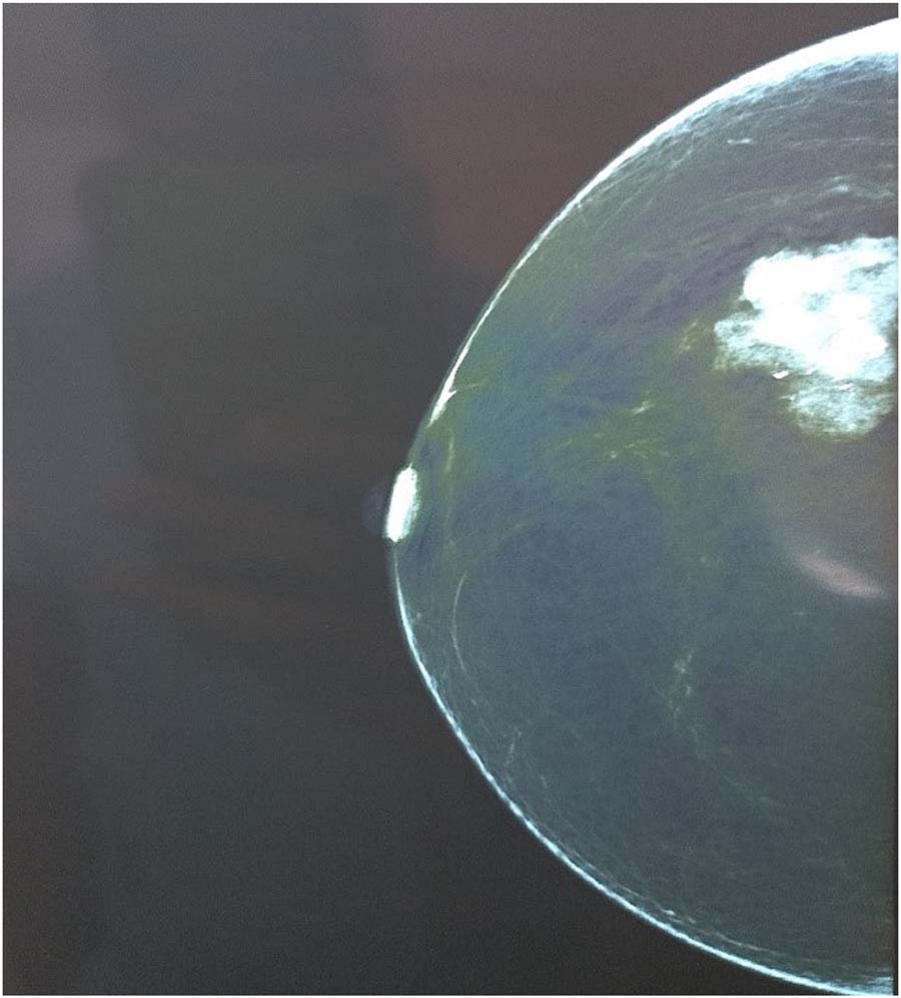
Mammogram of the right breast showing a 25 mm poorly defined opacity located at the junction of the outer quadrants, with architectural distortion but no microcalcifications, illustrating the radiologic appearance of adenoid cystic carcinoma of the breast.

A mammogram (Fig. 1) was performed, revealing a 25 mm poorly defined opacity in the outer quadrants of the right breast. Architectural distortion but no microcalcifications, raising suspicion of malignancy, the tumor was classified as ACR 4b.

A tru-cut biopsy was performed, the histology suggesting a complex microglandular adenosis.

**Figure 2:**
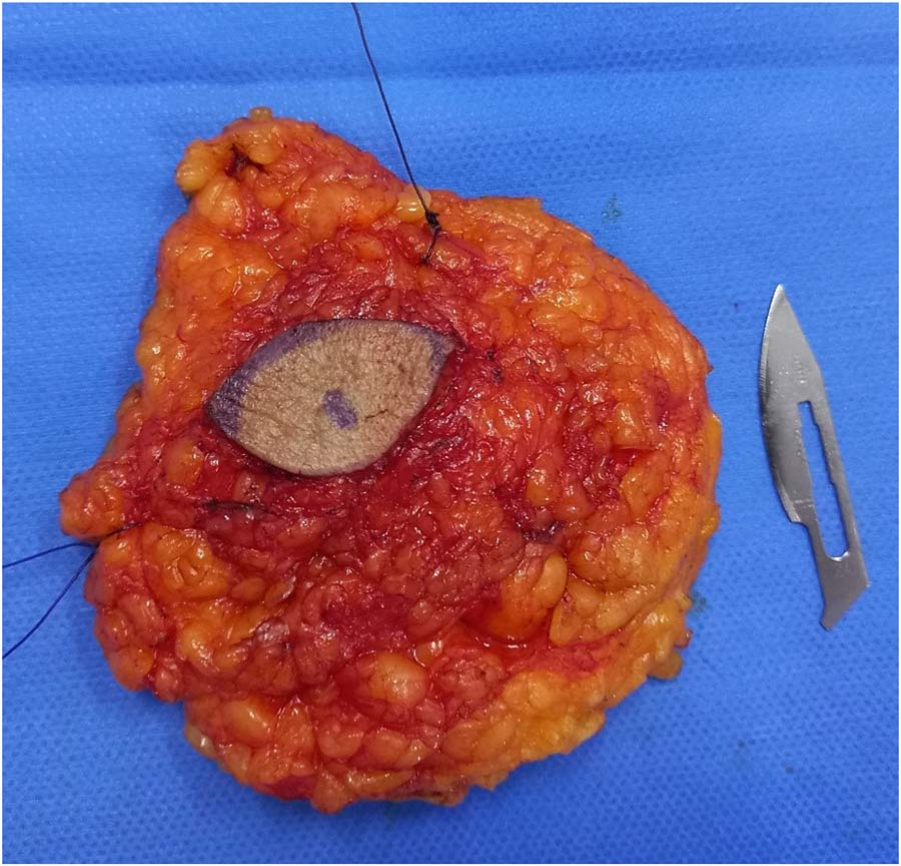
Gross photograph of the lumpectomy specimen showing a firm, grayish-yellow, poorly defined nodule measuring 3 × 2.5 × 3.1 cm, included to demonstrate the macroscopic features of the excised tumor.

An intraoperative frozen section examination was performed on a representative sample of the breast tumor for diagnostic purposes. The analysis revealed a malignant epithelial proliferation with glandular and tubular features, consistent with an invasive carcinoma. However, due to freezing artifacts and the architectural complexity of the lesion, definitive histological classification could not be established intraoperatively. In view of the tumor size and the diagnostic uncertainty at frozen section, the surgical team proceeded with axillary lymph node dissection (Fig. 2).

The lumpectomy specimen showed a firm, grayish-yellow, poorly defined nodule measuring 3 × 2.5 × 3.1 cm. Clear lateral margins, with the closest deep margin 5 mm from the tumor.

**Figure 3:**
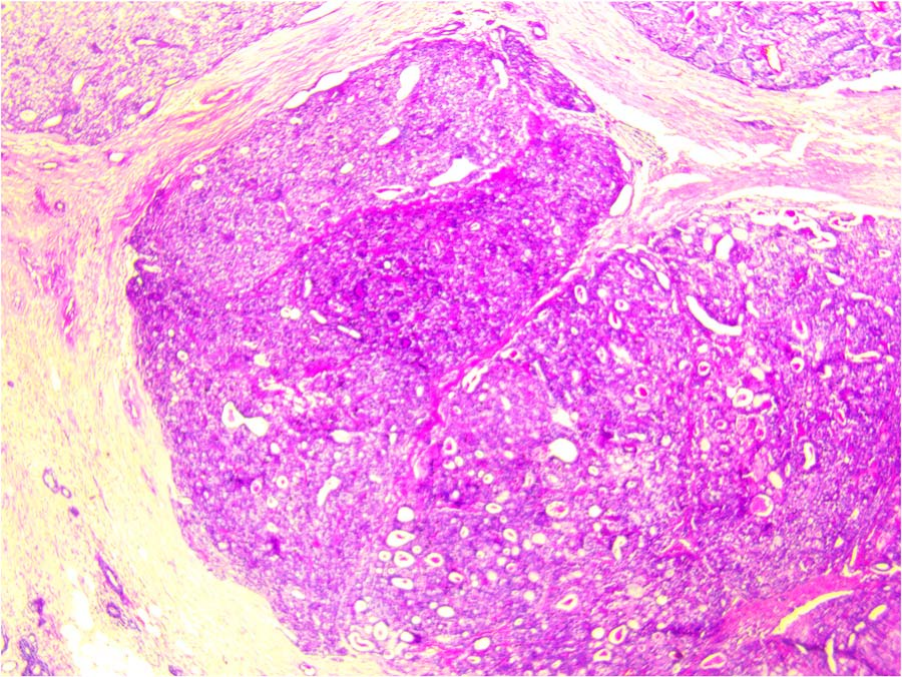
A typical cribriform architectural pattern composed of pseudocystic glandular spaces containing basophilic extracellular material.

Microscopic examination demonstrated a multinodular tubular pattern with cylindrical, mucin-filled spaces. The tumor displayed a characteristic dual-cell population, composed of inner basaloid cells with hyperchromatic nuclei and an outer layer of flattened myoepithelial cells forming a well-defined bilayered arrangement. The basement membrane appeared markedly thickened. Perineural invasion, a hallmark feature of adenoid cystic carcinoma (ACC), was identified. No solid growth pattern or vascular emboli were observed (Fig. 3).

The tumor was classified as Grade I according to the MD Anderson Cancer Center grading system.

**Figure 4:**
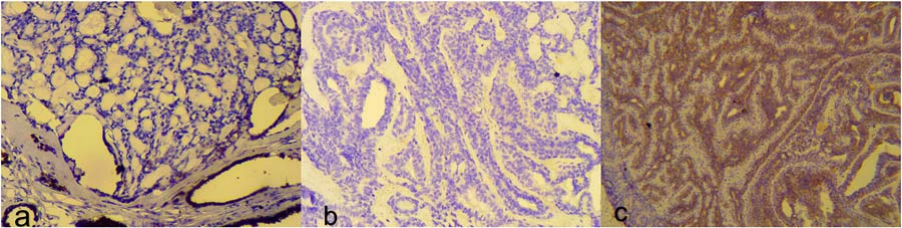
**a-c**: Histopathological and immunohistochemical features of adenoid cystic carcinoma (ACC) of the breast. (a) Immunohistochemical staining of hormone receptors (ER, PR). Estrogen receptor (ER) and progesterone receptor (PR) are not expressed in the tumor section. (b) Immunohistochemical staining of HER-2/neu. HER-2/neu is not expressed in the tumor section. (c) Immunohistochemical staining of CD117 (c-kit). CD117 shows strong cytoplasmic positivity in the tumor section.

Immunohistochemical analysis demonstrated that estrogen receptor (ER) and progesterone receptor (PR) were both negative, and HER-2/neu expression was absent. In contrast, CD117 (c-kit) showed strong cytoplasmic positivity, CK7 and pancytokeratin were positive. These findings confirm the triple-negative phenotype of the tumor. These results confirmed the triple-negative nature of the tumor. (Fig. 4a-c).

A staging thoracoabdominopelvic CT scan revealed no metastatic lesions, confirming localized disease. Although PET-CT is considered superior for comprehensive staging in oncology, it was not performed in this case due to limited availability in our healthcare setting.

The case was reviewed in a multidisciplinary team meeting. Based on consensus, adjuvant radiotherapy was indicated due to the tumor’s subtype and recurrence risk. The patient received 50 Gy to right whole breast 2 Gy/fraction, 5 days/week, with good tolerance and no significant toxicity. At one-year follow-up, the patient remains disease-free clinically and radiologically.

## Discussion

Adenoid cystic carcinoma (ACC), formerly known as cylindroma, is a distinct pathological entity first described in 1946. It is a malignancy that primarily affects the salivary glands and upper airways but can also be found in other anatomical sites such as the external auditory canal, Bartholin’s glands, and the uterine cervix [1, 2]. Its occurrence in the breast is extremely rare, representing only 0.1% to 1% of all breast cancers.

Breast ACC predominantly affects postmenopausal women and is characterized by slow growth and low malignant potential, unlike ACC in other locations, which tend to be more aggressive. While it predominantly affects women, cases have also been reported in men. The average age of diagnosis is 61 years [3, 5], with cases ranging from 19 to 94 years. The age distribution is generally similar to that of invasive ductal carcinoma (IDC).

The most common presenting symptom of breast ACC is a well-circumscribed, mobile, central breast nodule. Approximately 24% of patients report pain, which is attributed to perineural invasion by the tumor. Mammographic findings are non-specific and resemble other breast carcinomas, though the absence of microcalcifications is a distinguishing feature. This was consistent with the findings in our patient.

Histologically, breast ACC resembles ACC at other sites and is composed of two cellular components: small basaloid cells and epithelial cells. It exhibits variable architectural patterns, including glandular, trabecular, basaloid, cribriform, or solid formations. In our case, the tumor predominantly showed a tubular and cribriform pattern with abundant mucin-filled spaces. A dual cellular population was evident: inner basaloid cells with hyperchromatic nucleir and outer myoepithelial cells forming a distinct layer, separated by a thickened basement membrane. Immunohistochemically, in our case, the epithelial component was positive for CK7 and pancytokeratins. We did not specifically evaluate myoepithelial markers in the basaloid component. However, according to the literature, the basaloid component frequently expresses myoepithelial markers such as vimentin, smooth muscle actin, calponin, SMA and p63 [4, 6]. No solid areas or vascular emboli were identified. These features are consistent with low-grade ACC and explain its usually indolent clinical course.

Hormone receptor expression is consistently negative, and while HER-2/neu status in breast ACC has been infrequently studied, it is typically not expressed, as seen in this case. Breast ACC meets the diagnostic criteria for triple-negative basal-like breast cancer (ER-, PR-, HER-2-, CK5/6+, KIT+). However, its prognosis is significantly better than the triple-negative invasive ductal carcinoma (TNBC) [2–8].

The main differential diagnoses include intraductal carcinoma and infiltrating carcinoma, particularly the tubular or cribriform subtypes. Immunohistochemistry is crucial in confirming the diagnosis.

There is no standardized treatment protocol for breast ACC, mainly due to its rarity. Ro et al. [9] proposed a classification system based on the percentage of solid components in the tumor and suggested three therapeutic strategies:

Grade I (absence of solid elements): Lumpectomy.

Grade II (<30% solid elements): Mastectomy.

Grade III (>30% solid elements): Mastectomy with axillary lymph node dissection.

Histological involvement of axillary lymph nodes in adenoid cystic carcinoma of the breast is exceptionally rare, with a reported incidence ranging from 0.8% to 6.7% [7]. Therefore, routine axillary dissection is generally not recommended. However, in the present case, axillary dissection was performed due to the relatively large tumor size and the diagnostic uncertainty encountered during intraoperative frozen section analysis.

Adjuvant radiotherapy plays a role in local disease control, especially in cases of breast-conserving surgery.

Systemic adjuvant therapy is not standardized. Endocrine therapy is not indicated in triple-negative ACC. Multiple cohort studies indicate no clear benefit from adjuvant chemotherapy, even in higher-risk subgroups, whereas surgery plus RT achieves excellent 5-year survival (>90%) [12]. Cytotoxic therapy can be reserved for exceptional high-grade or solid-variant ACC, positive margins not amenable to re-excision, nodal/distant disease, or participation in clinical trials. Targeted approaches (e.g. c-KIT) remain investigational [2, 3, 5].

Regarding staging, major guidelines do not recommend routine baseline FDG-PET/CT for early, operable breast cancer; PET/CT may be considered only when conventional imaging is inconclusive or for higher-stage disease [10, 11]. In our setting, staging with contrast-enhanced CT verified localized disease and, given resource constraints and the lack of an evidence-based indication, PET/CT was not pursued. This approach aligns with value-based recommendations and the realities of care in resource-limited systems.

Breast ACC is classified as a low-grade malignancy with a favorable prognosis, significantly better than ACC in salivary glands or the upper airways. However, late recurrences and distant metastases can occur, even more than 15 years after treatment, necessitating long-term follow-up [2, 6, 8].

Post-treatment surveillance should include regular clinical and radiological assessments. Annual mammography is recommended to detect local recurrence after breast-conserving therapy and to monitor the contralateral breast. In addition, periodic chest radiography may be performed to screen for late pulmonary metastases.

## Conclusion

Adenoid cystic carcinoma of the breast is a rare histological entity that primarily affects postmenopausal women. Its clinical presentation and radiological features are non-specific, making diagnosis challenging. While its histological characteristics are distinctive, it can be misdiagnosed as other types of breast carcinoma, highlighting the importance of immunohistochemical analysis.

There is no standardized treatment protocol, however breast ACC has an exceptionally favorable prognosis. However, due to the risk of late local or distant recurrences, long-term follow-up is essential to ensure early detection and appropriate management of potential recurrences.
